# Multi-modal Prehabilitation and Outcomes after Gastroesophageal Cancer Surgery: A Cohort Study of Pulmonary Complications and Resilience to Major Postoperative Events

**DOI:** 10.1245/s10434-026-19934-1

**Published:** 2026-07-01

**Authors:** David S. Liu, Khang Duy Ricky Le, Jaimee Cacic, Bianca Ukovic, Christie Mellerick, Blake Binion, Greta Ishak, Jesse Turner, Carlene Wilson, Liam Johnson, Neha Suryanarayan, Laurence Weinberg, Marissa Ferguson, Celia Lanteri, Danny Brazzale, Ronald Ma, Amanda Dalyell, Katheryn Hall, Linda Watson, Brooke Chapman, Benjamin Keong, Krinal Mori, Ahmad Aly, Darren Wong, Ashley Bigaran

**Affiliations:** 1 Department of Surgery, University of Melbourne, Heidelberg, VIC Australia; 2 Upper Gastrointestinal Surgery Unit, Division of Surgery, Anesthesia and Procedural Medicine, Austin Health, Heidelberg, VIC Australia; 3 Victorian Interventional Research and Trials Unit, Department of Surgery, University of Melbourne, Austin Precinct, Heidelberg, VIC Australia; 4 Division of Cancer Surgery, Peter MacCallum Cancer Centre, Parkville, VIC Australia; 5 Department of General Surgical Specialties, The Royal Melbourne Hospital, Parkville, Melbourne, VIC Australia; 6 Faculty of Health, School of Medicine, Deakin University, Geelong, Melbourne, VIC Australia; 7 Department of Nutrition and Dietetics, Austin Health, Melbourne, VIC Australia; 8 Wellness and Supportive Care, Olivia Newton-John Cancer Research and Wellness Centre, Austin Health, VIC, Australia; 9 School of Exercise and Nutrition Sciences, Deakin University, Burwood, VIC Australia; 10 School of Behavioral Sciences, Australian Catholic University, Melbourne, VIC Australia; 11 Department of Psychology, Allied Health Directorate, Austin Health, Heidelberg, VIC Australia; 12 Department of Anesthesia, Austin Health, Melbourne, VIC Australia; 13 Department of Critical Care, the University of Melbourne, VIC, Australia; 14 Department of Respiratory Medicine, Austin Health, Heidelberg, VIC Australia; 15 Health Information Systems, Austin Health, Melbourne, VIC Australia; 16 Department of Surgery, Northern Hospital, Epping, VIC Australia; 17 Department of Gastroenterology, Austin Health, Heidelberg, VIC Australia; 18 Clinical Innovations Laboratory, Olivia Newton-John Cancer Research Institute, Heidelberg, VIC Australia

**Keywords:** Prehabilitation, Esophageal cancer, Gastric cancer, Enhanced prehabilitation, Perioperative optimization

## Abstract

**Background:**

Esophago-gastric cancer surgery frequently results in substantial morbidity. Prehabilitation, an approach to optimize an individual’s preoperative physical, nutritional, and mental health, may decrease postoperative complications. Here, we present perioperative and allied health findings from a multimodal prehabilitation study (ACTRN12623000183684).

**Methods:**

This single-center observational study recruited patients with esophago-gastric cancer undergoing curative-intent treatment between 2017 and 2024. Patients who did and did not receive multimodal prehabilitation were compared. Prehabilitation involved personalized medical, physical, nutritional, and psychological optimization before surgery. The primary endpoint was the rate of postoperative respiratory complications. Secondary endpoints included other postoperative complications and allied health outcomes.

**Results:**

In total, 164 participants (control *n*=121, prehabilitation *n*=43) underwent esophago-gastric cancer resection. More minimally invasive surgeries were performed in the prehabilitation group, but the baseline characteristics were otherwise similar between groups. Prehabilitation improved physical fitness and decreased malnutrition rates. Importantly, prehabilitation resulted in fewer postoperative respiratory (43.8% vs 20.9%, *p*=0.010), cardiac (22.3% vs 7.0%, *p*=0.037), hepatic (9.1% vs 0.0%, *p*=0.031), and renal (9.9% vs 0.0%, *p*=0.037) complications than in the control group. Moreover, prehabilitation was associated with diminished intensive care unit stays (median [IQR] 1 day [1.0–6.5] vs 1 day [1.0–3.0]; *p*=0.005) and hospital stays (14 days [9–24] vs 11 days [8–16]; *p*<0.001) and higher rates of adjuvant chemotherapy usage (51.0% vs 80.6%, *p*=0.004). Multivariate and sensitivity analyses demonstrated that prehabilitation, rather than surgical technique (laparoscopic vs open surgery), was independently associated with perioperative outcomes.

**Conclusion:**

This study demonstrates that multimodal prehabilitation improves perioperative outcomes for patients with esophago-gastric cancer, thus supporting routine integration of prehabilitation into perioperative care pathways.

**Supplementary Information:**

The online version contains supplementary material available at https://doi.org/10.1245/s10434-026-19934-1.

Esophago-gastric cancer surgery is associated with substantial morbidity. International data have indicated perioperative morbidity and mortality rates of 30–60% and 1–3%, respectively.^1,2^ Surgery, with or without neoadjuvant treatment, remains the cornerstone of curative-intent treatment for localized disease.^3,4^ Despite advances in surgical techniques, anesthesia, and enhanced recovery after surgery pathways, postoperative complications such as pulmonary or cardiac complications remain common.^2^ Although baseline functional status is a key determinant of surgical outcomes, many patients with esophago-gastric cancer have complex comorbidities, including frailty or cachexia, which are often worsened by neoadjuvant therapies.^5,6^ These adverse effects, together with surgical stress, may delay adjuvant systemic therapies, compromise cancer-specific survival, diminish quality of life, and increase healthcare resource use through prolonged hospital stays, intensive care, and hospital readmission.^7–13^ Therefore, a need exists to evaluate the efficacy of interventions that optimize physiological resilience to decrease surgical complications in patients preparing for esophago-gastric cancer surgery.

Multimodal prehabilitation before high-risk surgery, particularly in abdominal, cardiac, and non-cancer conditions, has markedly increased in recent years because of its favorable effects on enhancing a patient’s ability to withstand the physiological stress of major surgery.^14^ Multimodal prehabilitation encompassing exercise training, nutritional intervention, and psychological support has been reported to decrease postoperative complications in those undergoing esophago-gastric cancer surgery.^15,16^ Despite these promising findings, postoperative outcomes remain heterogeneous, largely because of inconsistent application of multimodal interventions across literature. Furthermore, evidence for guiding the design, assessment, timing, and delivery of multimodal prehabilitation is limited, particularly outside European and North American centers where esophago-gastric surgery is typically centralized to high-volume institutions.

Accordingly, this study aimed to evaluate the effects of a structured multimodal prehabilitation program encompassing medical, physical, nutritional, and psychological optimization on allied health and perioperative outcomes in patients undergoing esophago-gastric cancer surgery at an Australian tertiary hospital.

## Methods

### Study Design

This single-center study prospectively recruited patients with esophago-gastric cancer in 2022–2024 who were undergoing curative-intent treatment and consented to participate in a multimodal prehabilitation program. This cohort was compared with consecutive patients with esophago-gastric cancer in 2017–2024 who received curative-intent treatment by the same treating team but received no prehabilitation, either because they declined to participate or they were treated before our prehabilitation program existed. This study was registered with the Australian New Zealand Clinical Trials Registry (ACTRN12623000183684) and approved by the Austin Health Human Research Ethics Committee (HREC/88598/Austin-2022).

### Participants

Eligible participants were adults aged ≥18 years with gastroesophageal adenocarcinoma or squamous cell carcinoma who were scheduled to undergo elective esophagectomy or gastrectomy with or without perioperative therapies. The exclusion criteria included patients with stage 4 cancer, non-curative treatment, no surgery, and local tumor excision without radical lymphadenectomy.

### Interventions

The full methodological detail related to the study interventions is outlined in the Supplementary Material. The clinically integrated prehabilitation program included medical, physical, nutritional, and psychological support. A 4-week prehabilitation program was delivered to patients who underwent upfront surgery. For those who received neoadjuvant therapy before surgery, a 3-month program was delivered. The full methodological details of each aspect of this multimodal prehabilitation program are provided in the Supplementary Material and briefly described below. Each participant commenced prehabilitation as soon as the intent for cure was established after multidisciplinary tumor board meetings.

#### Exercise Training Program

This program combined supervised aerobic and resistance exercise training (45–60 min twice each week) with additional unsupervised aerobic exercise training (60 min/week). Aerobic exercise training progressed from moderate-intensity continuous training to high-intensity interval training, as quantified with a baseline cardiopulmonary exercise test (CPET).^17–19^ Resistance exercise training targeted major muscle groups (~60% of one-repetition maximum, 2–4 sets, and maximum 8–12 repetitions) and progressed accordingly.^20^ Heart rate and ratings of perceived exertion were monitored throughout the intervention.^21^

#### Nutritional Intervention

Participants were assessed by an experienced upper gastrointestinal cancer surgery dietitian at baseline, during neoadjuvant treatment, and before surgery. Individualized nutrition advice was aimed at addressing nutrition-impact symptoms, limiting nutritional decline during neoadjuvant therapy, implementing oral or enteral nutrition support, and enhancing physiological reserve before surgery. All participants in both the prehabilitation and the control groups received immune-modulating supplementation (IMPACT, Nestle Australia Ltd®, 2×250 mL) for 5 days pre- and postoperatively.

#### Psychological Support

Psychological support focused on distress management, adherence to the prehabilitation program, and coping with cancer treatment. Screening was conducted with the Hospital Anxiety and Depression scale (data not shown), with clinical psychology involvement, as indicated (when the Hospital Anxiety and Depression survey score was ≥8).

#### Medical Optimization and Anesthetic Support

Preoperative anesthetic assessment included a comprehensive medical history, CPET, and other diagnostic tests, as clinically indicated. Optimization strategies included specialist referrals, smoking cessation, multidisciplinary coordination, and perioperative pain management planning, including regional or neuraxial blocks.

### Outcome Measures and Definitions

The full methodological detail for this study is outlined in the Supplementary Material. Data were collected through an online REDCap registry embedded within our surgical unit. A research nurse conducted data cleaning. The primary endpoint was overall postoperative respiratory complications, as defined by the Esophagectomy Complications Consensus Guidelines.^22^ Secondary perioperative outcomes included individual complication rates according to the same guideline definitions, American Society of Anesthesiologists (ASA) score at time of surgery, highest Clavien–Dindo complication grade, durations of intensive care unit (ICU) and hospital stay, achievement of textbook outcome criteria, days alive and at home within 30 days after surgery (DAH30), initiation of postoperative chemotherapy,^23–26^ and 30-day reintervention, hospital and ICU readmission, and mortality rates. Cardiorespiratory fitness was assessed with CPET for the peak oxygen uptake (VO₂_peak_; mL/kg/min and L/min), ventilatory threshold, minute ventilation/carbon dioxide (VE/VCO₂) slope, resting and peak blood pressure, heart rate, peak power output, and respiratory exchange ratio.^17^ The 6-minute walk distance was also assessed as a measure of cardiorespiratory fitness.^27^ Functional muscle strength was measured with handgrip dynamometry,^28,29^ and the 30-second sit-to-stand test was used to assess lower limb endurance. Anthropometrics included weight, height, body mass index, and skinfold/mid-arm/calf measurements.^23–26^ Energy expenditure (kcal/day) was measured with a Fitmate GS^®^ indirect calorimeter that calculates the amount of exhaled carbon dioxide (VCO_2_) and consumed oxygen (VO_2_).^28^

### Sample Size Calculation

Given the anticipated ratio of 2:1 for control versus prehabilitation participants, 180 patients (control: *n*=120, prehabilitation: *n*=60) were determined to be required to detect a 20% absolute difference in overall respiratory complication rate between groups with 80% power (α<0.05). Allowances were made *a priori* for interim analyses and for the power calculation to be revised as needed. After the last planned interim analysis (43rd participant in the prehabilitation group), the study steering committee decided to end the trial early because the primary endpoint of efficacy had been met.

### Statistical Analysis

Univariate comparisons between study groups were performed with Fisher’s exact test for categorical variables and Student’s t-test for continuous variables. For non-parametric continuous data, the Mann–Whitney U test was used. For comparisons involving more than two categorical variables, the chi-squared test was applied. Multivariate analysis of the primary outcome was conducted with logistic regression. Baseline variables with *p*<0.200 in the univariate comparison were included in multivariate modelling. A sensitivity analysis was conducted for the minimally invasive surgery subgroup, to validate findings of the main analysis. Two-tailed P values <0.050 and odds ratios (ORs) with 95% confidence intervals (CIs) that did not cross 1 were considered statistically significant. Statistical analyses were conducted in Prism version 10.2.2 (GraphPad Software, San Diego, CA, USA)

## Results

### Patient Characteristics

A total of 164 participants with esophago-gastric cancer were included (Table 1), comprising 43 participants who underwent multimodal prehabilitation and 121 controls. At baseline, no significant differences in age, sex, body mass index, Charlson comorbidity index, smoking status, or Eastern Cooperative Oncology Group performance status were observed between groups. Clinical tumor features and preoperative treatment pathways were comparable: 91 participants received neoadjuvant chemotherapy and 13 received chemoradiotherapy.^30,31^ Overall, 77 (47.0%) participants underwent esophagectomy, whereas total and subtotal gastrectomy were performed in 34 (20.7%) and 53 (32.3%) participants, respectively. A significant difference in surgical approach (open vs minimally invasive) was observed between groups. The higher rate of minimally invasive surgery in the prehabilitation group reflected temporal changes in surgical practice, because the control cohort included patients treated prior to 2022, before minimally invasive techniques became more common in Australia.

**Table 1:** Baseline characteristics

Characteristics	Control (*n*=121)	Prehabilitation (*n*=43)	*p*-value
*Demographics*			
Age, years	67.9 ± 9.8	67.2 ± 10.7	0.719
Sex, male	91 (75.2)	32 (74.4)	1.000
BMI, kg/m^2^	27.1 ± 5.6	26.1 ± 5.2	0.281
Charlson Comorbidity Index, score	4 (3–6)	4 (3–6)	0.247
Smoking status at time of surgery			0.380
Active	12 (9.9)	3 (7.0)	
Former	59 (48.8)	17 (39.5)	
Never	50 (41.3)	23 (53.5)	
ECOG performance status	0 (0–1)	0 (0–1)	0.500
*Clinical tumor features*			
Histological diagnosis			0.102
Adenocarcinoma	111 (91.7)	39 (90.7)	
Adenocarcinoma with squamous differentiation	0 (0.0)	1 (2.3)	
Squamous cell carcinoma	10 (8.3)	2 (4.7)	
Neuroendocrine tumor	0 (0.0)	1 (2.3)	
Anatomical location of tumor			0.287
Esophagus/gastroesophageal junction (Siewert 1 and 2)	71 (58.7)	21 (48.8)	
Stomach/gastroesophageal junction (Siewert 3)	50 (41.3)	22 (51.2)	
Clinical stage			0.215
cT1 N0 M0	25 (20.7)	13 (30.2)	
cT2–3 N0 M0	48 (39.7)	18 (41.9)	
cT4a N0 M0	4 (3.3)	0 (0.0)	
cT4b N0 M0	0 (0.0)	1 (2.3)	
cT1 N1 M0	2 (1.7)	0 (0.0)	
cT2–3 N1 M0	34 (28.1)	7 (16.3)	
cT4a N1 M0	8 (6.6)	4 (9.3)	
Preoperative treatment			0.911
None	45 (37.2)	15 (34.9)	
Chemotherapy (FLOT protocol)	66 (54.5)	25 (58.1)	
Chemoradiotherapy (CROSS protocol)	10 (8.3)	3 (7.0)	
*Surgery details*			
Type			0.560
Subtotal gastrectomy	39 (32.2)	14 (32.6)	
Total/extended total gastrectomy	23 (19.0)	11 (25.6)	
Trans-hiatal esophagectomy	2 (1.7)	0 (0.0)	
2-stage esophagectomy	39 (32.3)	15 (34.9)	
3-stage esophagectomy	18 (14.9)	3 (7.0)	
Approach			**<0.001**
Minimally invasive	47 (38.8)	36 (83.7)	
Open	74 (61.2)	7 (16.3)	
Lymphadenectomy			0.591
2-field mediastinal and abdominal	59 (48.8)	18 (41.9)	
D1 abdominal	8 (6.6)	2 (4.7)	
D1+ abdominal	54 (44.6)	19 (53.5)	
Lymph node yield	31 (23–46)	35 (24–47)	0.831
Intraoperative complications	4 (3.3)	2 (4.7)	0.653
Surgical duration, minutes	379.2 ± 128.0	395.9 ± 127.9	0.463
Epidural anesthesia use	57 (47.1)	13 (30.2)	0.072

Data are presented as mean ± standard deviation, n (%) or median (interquartile range) unless otherwise indicated. BMI, body mass index; ECOG, Eastern Cooperative Oncology Group performance status grading.

### Pulmonary Complications

Compared with controls, the prehabilitation group had a significantly lower overall rate of pulmonary complications (control 43.8% vs prehabilitation 20.9%, *p*<0.010, Table 2). Specifically, the rates of pneumonia (28.1% vs 11.6%, *p*=0.036) and atelectasis requiring intervention (23.1% vs 2.3%, *p*<0.001) were significantly lower in the prehabilitation group. Multivariate analysis controlling for baseline confounders, including surgical approach, confirmed prehabilitation as an independent predictor of fewer postoperative complications (OR 0.22; 95% CI 0.08–0.55, *p*=0.002) than observed in the control group (Table S3).

**Table 2:** Pulmonary outcomes

Pulmonary complications	Control (*n*=121)	Prehabilitation (*n*=43)	OR (95% CI)	p-Value
All pulmonary complications	53 (43.8)	9 (20.9)	0.34 (0.14–0.78)	0.010
Pneumonia	34 (28.1)	5 (11.6)	0.34 (0.13–0.88)	0.036
Atelectasis requiring intervention	28 (23.1)	1 (2.3)	0.08 (0.01–0.45)	0.001
Pleural effusion requiring drainage	15 (12.4)	2 (4.7)	0.34 (0.08–1.38)	0.243
Respiratory failure requiring intubation	5 (4.1)	1 (2.3)	0.55 (0.05–4.26)	1.000
Pneumothorax requiring intervention	4 (3.3)	0 (0.0)	0.00 (0.00–2.88)	0.574
Acute respiratory distress syndrome	3 (1.7)	0 (0.0)	0.00 (0.00–3.25)	0.568
Acute aspiration	6 (5.0)	3 (7.0)	1.44 (0.38–5.37)	0.699
Tracheobronchial injury	2 (1.7)	0 (0.0)	0.00 (0.00–6.10)	1.000

Data are presented as n (%) unless otherwise indicated. CI, confidence interval; OR, odds ratio; SD, standard deviation.

#### Perioperative Outcomes

Perioperative outcomes between groups are summarized in Tables 3 and 4. Clavien–Dindo complication grading indicated that prehabilitation was associated with fewer minor (grade 1 and 2) complications (control 44.6% vs prehabilitation 14.0%, *p*<0.001), whereas no difference was observed in major (grade ≥3) complications (31.4% vs 37.2%, *p*=0.571) between groups (Table 3). Moreover, prehabilitation was associated with lower rates of overall cardiac (22.3% vs 7.0%, *p*=0.037), liver (9.1% vs 0.0%, *p*=0.031), and acute kidney injury (9.9% vs 0.0%, *p*=0.037) complications than observed in the control group (Table 3). Compared with the control group, the prehabilitation group had more favorable ASA grading at the time of surgery (median [interquartile range (IQR)] 3 [3] vs 3 [2, 3] grade, *p*=0.003), shorter ICU stays (1 day [1–6.5] vs 1 day [1–3], *p*=0.005) and hospital stays (14 days 9–24] vs 11 days [8–16], *p*<0.001); these improvements were associated with longer DAH30 (15 days [2–21] vs 18 days [13–22], *p*=0.023) and higher rates of adjuvant therapy use (51.0% vs 80.6%, *p*=0.004, Table 4).

**Table 3:** Esophagectomy complication consensus guidelines complication profile

Complications	Control (*n*=121)	Prehabilitation (*n*=43)	OR (95% CI)	*p*-Value
Clavien–Dindo complication grade			-	**0.004**
No complications	29 (24.0)	21 (48.8)		
Grade 1	20 (16.5)	2 (4.7)		
Grade 2	34 (28.1)	4 (9.3)		
Grade 3 a/b	28 (23.1)	11 (25.6)		
Grade 4 a/b	7 (5.8)	5 (11.6)		
Grade 5	3 (2.5)	0 (0.0)		
Minor complications, grade 1 and 2	54 (44.6)	6 (14.0)	0.20 (0.08–0.50)	**<0.001**
Major complications, grade ≥3	38 (31.4)	16 (37.2)	1.29 (0.62–2.71)	0.571
*Cardiac*				
All cardiac complications	27 (22.3)	3 (7.0)	0.26 (0.08–0.82)	**0.037**
Arrhythmias requiring intervention	23 (19.0)	3 (7.0)	0.32 (0.10–1.04)	0.088
Congestive heart failure requiring intervention	8 (6.6)	0 (0.0)	0.00 (0.00–1.27)	0.112
Myocardial infarction	1 (0.8)	0 (0.0)	0.00 (0.00–25.33)	1.000
Cardiac arrest requiring intervention	0 (0.0)	0 (0.0)	-	1.000
Pericarditis requiring treatment	0 (0.0)	0 (0.0)	-	1.000
*Gastrointestinal*				
Anastomotic leak	18 (14.9)	3 (7.0)	0.43 (0.13–1.47)	0.287
Conduit necrosis	1 (0.8)	0 (0.0)	0.00 (0.00–25.33)	1.000
Ileus delaying enteral feeding	12 (9.9)	9 (20.9)	2.40 (0.99–6.07)	0.107
Small bowel obstruction	3 (2.5)	2 (4.7)	1.92 (0.33–9.62)	0.607
Chyle leak	7 (5.8)	3 (7.0)	1.22 (0.33–4.75)	0.723
Pancreatitis	1 (0.8)	2 (4.7)	5.85 (0.66–85.37)	0.169
Liver dysfunction	11 (9.1)	0 (0.0)	0.00 (0.00–0.81)	**0.031**
Acute diaphragmatic hernia	0 (0.0)	1 (2.3)	0.00 (0.00–3.20)	0.262
*Infective*				
General sepsis	3 (2.5)	1 (2.3)	0.94 (0.07–6.43)	1.000
Surgical site infection requiring intervention	8 (6.6)	1 (2.3)	0.33 (0.03–2.34)	0.448
*Clostridioides difficile* infection	1 (0.8)	0 (0.0)	0.00 (0.00–25.33)	1.000
Line infection requiring intervention	1 (0.8)	0 (0.0)	0.00 (0.00–25.33)	1.000
Intrathoracic abscess	3 (2.5)	3 (7.0)	2.95 (0.55–12.93)	0.186
Intraabdominal abscess	3 (2.5)	1 (2.3)	0.94 (0.07–6.43)	1.000
*Neurological*				
Delirium	7 (5.8)	2 (4.7)	0.79 (0.16–3.85)	1.000
Cerebrovascular accident	0 (0.0)	0 (0.0)	-	1.000
*Hematological*				
Bleeding requiring intervention	4 (3.3)	1 (2.3)	0.70 (0.06–4.41)	1.000
Venous thromboembolism	5 (4.1)	2 (4.7)	1.13 (0.22–5.61)	1.000
*Urological*				
Acute kidney injury	12 (9.9)	0 (0.0)	0.00 (0.00–0.72)	**0.037**
Urinary tract infection	3 (2.5)	0 (0.0)	0.00 (0.00–3.25)	0.568
*Skin*				
Wound dehiscence	2 (1.7)	0 (0.0)	0.00 (0.00–6.10)	1.000
Acute abdominal wall hernia	0 (0.0)	1 (2.3)	0.00 (0.00–3.20)	0.262

Data are presented as n (%) unless otherwise indicated. CI, confidence interval; OR, odds ratio.

**Table 4:** Perioperative outcomes

Perioperative outcomes	Control (*n*=121)	Prehabilitation (*n*=43)	OR (95% CI)	*p*-Value
ASA grade at time of surgery	3 (3–3)	3 (2–3)	-	**0.003**
Duration of ICU stay, days	1 (1–6.5)	1 (1–3)	-	**0.005**
Duration of hospital stay, days	14 (9–24)	11 (8–16)	-	**<0.001**
30-day reintervention	30 (24.8)	14 (32.6)	1.46 (0.71–3.02)	0.324
30-day ICU readmission	23 (19.0)	8 (18.6)	0.97 (0.41–2.26)	1.000
30-day hospital readmission	11 (9.1)	4 (9.3)	1.03 (0.34–3.17)	1.000
30-day mortality	3 (2.5)	0 (0.0)	0.00 (0.00–3.25)	0.568
Achieved textbook outcome criteria	38 (31.4)	18 (41.9)	1.57 (0.78–3.26)	0.262
DAH30, days	15 (2–21)	18 (13–22)	-	**0.023**
Received adjuvant treatment^a^	53 (51.0)	25 (80.6)	4.01 (1.58–10.26)	**0.004**

Data are presented as median (interquartile range) or n (%) unless otherwise indicated. ASA, American Society of Anesthesiologists; CI, confidence interval; DAH30, days alive and at home within 30 days after surgery; ICU, intensive care unit; OR, odds ratio. ^a^Clinically indicated to receive adjuvant treatment (control *n*=104, Strong for Oesophago-gastric Cancer Surgery prehabilitation *n*=31).

#### Sensitivity Analysis of Patients Who Underwent Minimally Invasive Surgery

To address the higher use of minimally invasive surgery in the prehabilitation group and its potential confounding effects, we performed a sensitivity analysis including only patients who underwent minimally invasive surgery (controls *n*=47; prehabilitation *n*=36, Table S4). In agreement with the primary analysis, prehabilitation was associated with significantly lower rates of pulmonary (control 53.2% vs prehabilitation 19.4%, *p*=0.003), cardiac (29.8% vs 5.6%, *p*=0.006), hepatic (10.6% vs 0.0%, *p*=0.044), and renal (10.6% vs 0.0%, *p*=0.044) complications than observed in the control group. These improvements were accompanied by significantly shorter ICU stays (median [IQR] 1 day [0–7] vs 1 day [1–3], *p*=0.042) and hospital stays (13 days [8–26] vs 11 days [8–16], *p*=0.009) and greater use of adjuvant therapy (45.5% vs 81.5%, *p*=0.007) than the control group.

#### Effects of Prehabilitation on Major Postoperative Complications

To examine the effects of prehabilitation on patients with major (Clavien–Dindo grade ≥3) postoperative complications, we analyzed their adverse event profiles and outcomes. Although both groups experienced a range of adverse events (Figure S1), the prehabilitation group had fewer intercurrent complications per patient (median [IQR] 3 [2–5] vs 2 [1–3]; *p*=0.022) (Table 5) than the control group. Moreover, compared with controls, patients who received prehabilitation and experienced a major postoperative complication had shorter stays in the ICU (median [IQR] 7 days [2–14] vs 3 days [1–6], *p*=0.002) and hospital (29 days [19–49] vs 16 days [13–25], *p*<0.001), as well as greater DAH30 (0 days [0–9] vs 14 days [4–16], *p*=0.002) and use of adjuvant therapy (22.2% vs 66.7%, *p*=0.011; Table 5).

**Table 5:** Perioperative outcomes in patients who experienced a major (grade ≥3) complication

Perioperative outcomes	Control (*n*=38)	Prehabilitation (*n*=16)	OR (95% CI)	*p*-Value
Intercurrent complications per patient	3 (2–5)	2 (1–3)	–	**0.022**
30-day reintervention	28 (73.7)	12 (75.0)	1.07 (0.30–3.58)	1.000
30-day ICU readmission	20 (52.6)	8 (50.0)	0.90 (0.30–2.68)	1.000
Duration of ICU stay, days	7 (2–14)	3 (1–6)	–	**0.002**
Duration of hospital stay, days	29 (19–49)	16 (13–25)		**<0.001**
30-day hospital readmission	7 (18.4)	2 (12.5)	0.63 (0.12–2.85)	0.709
30-day mortality	3 (7.9)	0 (0.0)	0.00 (0.00–2.73)	0.547
DAH30, days	0 (0–9)	14 (4–16)	-	**0.002**
Received adjuvant treatment^a^	8 (22.2)	8 (66.7)	7.00 (1.60–23.09)	**0.011**

Data are presented as median (interquartile range) or n (%) unless otherwise indicated. CI, confidence interval; DAH30, days alive and at home within 30 days after surgery; ICU, intensive care unit; OR, odds ratio. ^a^Clinically indicated to receive adjuvant treatment (control *n*=36, Strong for Oesophago-gastric Cancer Surgery prehabilitation *n*=12).

#### Allied Health and Functional Outcomes

Allied health and functional outcomes (Table 6) were compared with each participant’s baseline measurements. The median attendance for the exercise and nutritional intervention was 80% (IQR 25–100) and 100%, respectively. Multimodal prehabilitation was associated with improvements in median daily total energy intake (median [IQR] baseline 6.9 MJ [5.0–7.3] vs post 8.0 MJ [7.5–9.3], *p*<0.001) and protein intake (68 g [55.0–75] vs 82 g [75–100], *p*<0.001). There was a non-significant increase in absolute hand grip strength from 30 kg (27–37) to 32 kg (29–39) (*p*=0.300); however, when adjusted for age and sex, handgrip strength increased significantly for prehabilitation participants (*p*=0.034). These improvements were accompanied by a decrease in the proportion of patients with malnutrition from 42% at baseline to 14% post-prehabilitation (*p*=0.019). No significant changes were observed in mid–upper arm circumference, triceps skinfold, or calf circumference. Prehabilitation also improved the 6-minute walk distance (mean ± standard deviation [SD] 502 ± 92 vs 538 ± 104 m, *p*=0.017) and sit-to-stand repetitions (16 ± 5 vs 18 ± 7 repetitions, *p*=0.044). Moreover, VO_2peak_ (mL/kg/min) and other CPET-derived indices did not significantly change (all *p*>0.05).

**Table 6:** Nutritional and physical outcomes

Parameters	Baseline (*n*=43)	Post-prehabilitation (*n*=43)	*p*-Value
*Nutritional outcomes*^*a*^			
Hand grip strength, kg	30 (27–37)	32 (29–39)	0.300
Normative hand grip strength, %	107 (88–124)	113 (95–126)	**0.003**
Daily energy intake, MJ	6.9 (5.0–7.3)	8.0 (7.5–9.3)	**<0.001**
Daily protein intake, g	68 (55–75)	82 (75–100)	**<0.001**
Mid-upper arm circumference, cm	29 (28.2–30.8)	29.6 (28.5–31.5)	0.400
Triceps skin fold, mm	12 (7–16)	14 (9–19)	0.300
Calf circumference, cm	36.9 (34.5–38.2)	37.8 (36.1–39.3)	0.150
Malnutrition rate (%)	42	14	**0.019**
*CPET outcomes*			
V̇O_2peak_, L/min	1.7 ± 0.5	1.7 ± 0.5	0.320
V̇O_2peak_, mL/kg/min	22.0 ± 5.3	20.8 ± 4.7	0.120
PPO, watts	130 ± 42	130 ± 40	0.800
HR_peak_, beats per minute	148 ± 22	150 ± 19	0.570
V_E_/VCO_2_	29.5 ± 4.0	30.3 ± 5.4	0.390
RER_peak_	1.2 ± 0.1	1.2 ± 0.1	0.570
SBP_peak_, mmHg	171 ± 27	168± 27	0.590
DBP_peak_, mmHg	84 ± 12	86 ± 14	0.730
*Physical function outcomes*			
Six-minute walk test distance, m	502 ± 92	538 ± 104	**0.017**
Sit to standing, repetitions	16 ± 5	18 ± 7	**0.044**

Data are presented as median (interquartile range) or mean ± standard deviation unless otherwise indicated. CPET, cardiopulmonary exercise test; DBP_peak_, peak diastolic blood pressure.; HR_peak_, heart rate peak; PPO, peak power output; RER, respiratory exchange ratio; SBP_peak_, peak systolic blood pressure; VE/VCO_2_, minute ventilation/carbon dioxide; V̇O_2peak_ (L/min), absolute peak volume of oxygen uptake; V̇O_2peak_ (ml/kg/min), body weight indexed peak volume of oxygen uptake. ^a^Nutritional outcomes for *n*=34 patients.

## Discussion

The key findings from this study are that (1) multimodal prehabilitation improved perioperative outcomes, decreased healthcare resource use, and increased the use of adjuvant anticancer therapy, (2) prehabilitation decreased minor postoperative complications and enabled patients to overcome major postoperative complications with less morbidity, and (3) multimodal prehabilitation increased participants’ nutritional and physical fitness before esophago-gastric cancer surgery.

The most pronounced effect of multimodal prehabilitation was observed in postoperative pulmonary complications: overall pulmonary complications, pneumonia, and atelectasis requiring intervention were significantly lower than in controls. Beyond pulmonary complications, multimodal prehabilitation was associated with fewer minor postoperative complications, including cardiac, hepatic, and renal issues. Therefore, concurrently improving cardiopulmonary conditioning, nutritional status, and metabolic reserve might confer broader systemic benefits rather than an isolated pulmonary effect. Our results are consistent with those from prior observational studies and meta-analyses reporting lower rates of pulmonary complications than in controls.^32–34^ Halliday et al.^33^ demonstrated fewer postoperative pneumonia events (26% vs 66%; OR: 0.23) after a 16-week multimodal prehabilitation intervention in patients with esophageal cancer than in propensity-matched controls;^33,35^ however, randomized controlled trials have demonstrated variable effects.^36^ Although this discrepancy across studies remains a notable area of interest, it might be attributable to known heterogeneity in study design, multimodal prehabilitation intervention characteristics, intervention duration, and adherence.^36,37^ Our study adds to the evidence base indicating that multimodal prehabilitation decreases the surgical morbidity burden, even in patients with major postoperative complications; consequently, prehabilitation might mitigate further morbidity when severe complications occur.

In agreement with the favorable changes in postoperative complications, the physiological and clinical benefits of multimodal prehabilitation appeared to translate to improved perioperative outcomes, including more favorable pre-surgery ASA grading, shorter ICU and hospital lengths of stay, and greater DAH30. These perioperative results were consistent with observational data^33–35^ indicating that greater emphasis on physiological resilience before surgery leads to earlier discharge and faster functional recovery. Furthermore, this study extended prior observational evidence^33,35^ by demonstrating that multimodal prehabilitation not only improved the use/tolerance of neoadjuvant chemotherapy/radiation therapy but also translated to greater use of adjuvant therapy after surgery. Importantly, multimodal prehabilitation might have enabled more participants to experience fewer complications, thereby enhancing their ability to recover and proceed with systemic therapy. This oncologic outcome is critical, because failure to receive planned adjuvant therapy might be associated with poor survival.^38,39^ Participation in multimodal prehabilitation reinforces the oncological relevance beyond short-term surgical outcomes.

This study found that prehabilitation improved handgrip strength, dietary intake (energy and protein), 6-minute walk distance, lower limb endurance, and malnutrition rates. These improvements increased participants’ functional reserve to withstand the stress of major surgery and enhance recovery, thereby supporting diminished complications and subsequent lengths of stay, and elevated the ability for individuals to participate in post-surgical chemotherapy and radiotherapy.^40^ In contrast, no significant changes in CPET variables, such as VO₂peak, were observed. These mixed findings are consistent with broader evidence in esophago-gastric cancer cohorts.^41^ Potential explanations for this might include prehabilitation program heterogeneity, small sample sizes, and the deleterious effects of neoadjuvant chemotherapy on patient physiology.^42^ Given that VO_2peak_ is expected to decline by 10–20% during a 6-week neoadjuvant chemotherapy period,^37,43^ preservation appears to be the most consistent outcome across the scientific literature.^44^ Our results therefore highlight the importance of enhanced prehabilitation in maintaining the nutritional and physical capacity of patients undergoing these therapies.^40^

We acknowledge several limitations of this study. First, the non-randomized design and use of a historically enriched control cohort introduce potential biases that cannot be fully addressed by multivariable adjustment and sensitivity analyses. Secular trends in surgical practice, perioperative care, ICU admission thresholds, and oncologic management over 2017–2024 may have contributed to the observed differences. We attempted to mitigate confounders by including consecutive controls managed by the same surgical team, adjusting for baseline covariates, and performing subgroup analyses among patients undergoing minimally invasive surgery; nevertheless, residual confounding remains likely. Second, the prehabilitation cohort comprises patients who consented to and were able to participate in a structured program, raising the possibility of selection bias related to motivation, social support, or geographic proximity that may also influence outcomes. Third, the sample size was smaller than originally planned, and the study was stopped early by the steering committee after an interim analysis showed benefit for the primary endpoint. As no formal group-sequential stopping rules or alpha adjustments were applied, the magnitude of the treatment effect on respiratory complications and other outcomes may be overestimated, and our findings should be interpreted with appropriate caution. Fourth, we evaluated multiple secondary outcomes without adjustment for multiplicity, increasing the risk of type I error; the consistency of direction and biological plausibility across related endpoints partly mitigates this concern but does not eliminate it. Fifth, allied health and functional outcomes could only be compared within the prehabilitation group because comparable measures were not routinely collected in controls, limiting our ability to quantify between-group differences in these domains. Finally, this program was delivered at a single tertiary center with access to CPET, specialist dietitians, exercise physiologists, and clinical psychologists, which may limit generalizability to other healthcare settings.

Despite these limitations, our findings support the integration of multimodal prehabilitation into the perioperative care pathway for patients undergoing esophago-gastric cancer surgery. This has occurred at our hospital. Future research should prioritize adequately powered randomized trials that standardize multimodal interventions, incorporate robust adherence and fidelity metrics, and include longer-term oncologic endpoints such as disease-free and overall survival. Pragmatic implementation studies and cost-effectiveness analyses are also needed to define scalable models of care across diverse health systems. In the interim, our data suggest that prehabilitation can reduce both minor and organ-specific complications and, importantly, curtail the morbidity burden associated with major postoperative complications while enabling more patients to receive potentially curative adjuvant therapy.

## Conclusion

This study demonstrated that a multimodal prehabilitation program improved perioperative outcomes for patients after esophago-gastric cancer surgery. Our study further supports the routine integration of multimodal prehabilitation into perioperative care pathways.

## Supplementary Information

Below is the link to the electronic supplementary material.

Supplementary file1 (DOCX 166 kb)
