# Supplementary material for: Multi-modal Prehabilitation and Outcomes after Gastroesophageal Cancer Surgery: A Cohort Study of Pulmonary Complications and Resilience to Major Postoperative Events
Source: Ann Surg Oncol. 2026 Jul 1;33(12):10259–69. doi: 10.1245/s10434-026-19934-1 (PMC13675315; doi:10.1245/s10434-026-19934-1)
Supplement: Supplementary file 1 [file 10434_2026_19934_MOESM1_ESM.docx]

**SUPPLEMENTARY MATERIAL**

The Study Protocol detailing the full methodological detail of the prehabilitation program and can be found.

**Multimodal prehabiltiation intervention**

**Exercise intervention**

A summary of the exercise intervention is outlined in **Table 1**. A three-month optimized aerobic and resistance exercise training intervention, including high-intensity interval training, was prescribed. All exercise sessions were approximately 45 to 60 minutes and supervised by an accredited exercise physiologist or physiotherapist. Each participant was asked to complete additional, unsupervised exercise training, for at least 60 minutes per week to meet the current exercise guidelines ^1^ (not recorded).

**Aerobic exercise training**

The optimized aerobic exercise training intervention, including high-intensity interval training (HIIT), comprised of three target heart zone training zones, 1) moderate continuous exercise training (approximately 1-20 bpm below ventilatory threshold [VT]), 2) VT, and 3) HIIT (>95% peak heart rate achieved on the CPET) ^2,3^. The exercise training intensities was derived from each participant’s cardiopulmonary exercise test (VT). The first exercise training phase (weeks one to four) focused on improving exercise capacity (aerobic endurance). Participants performed two 30-minute moderate continuous exercise training sessions per week. In the second phase (weeks five to eight), participants performed a moderate continuous training session and either a VT or HIIT session depends on the participant’s exercise tolerability. The VT exercise session consisted of 20 minutes of continuous training at approximately 85% of their VO_2_peak. The high-intensity intervals began with low volume HIIT, which includes one minute of high-intensity exercise at >95% of peak heart rate, followed by one minute of active recovery at 60-75% of peak heart rate, repeated six to eight times. Moderate HIIT intervals included two to three minutes of high-intensity exercise at >95% of peak heart rate, followed by the equivalent timeframe for recovery (two- or three-minutes recovery) at 60-75% of peak heart rate, repeated four times. The final HIIT interval consisted of four minutes of high-intensity exercise >95% of peak heart rate followed by three minutes of active recovery at 60-75% of peak heart rate, repeated four times. In the final phase (weeks nine to 12), participants performed two HIIT sessions to align with the primary outcome twice weekly. All participants wore heart rate monitors throughout each training session for safety.

**Resistance training**

Participants performed two whole-body progressive resistance exercise training sessions per week based on functional status that included but not limited to upper and lower body resistance exercises. The initial intensity was set at 60% of one-repetition maximum at two to four sets of 12 to 8 repetitions maximum for 12 weeks ^1^. Progressive overload was applied throughout the exercise intervention and monitored by decrement changes in the rating of perceived exertion (change from the previous week by two decrement points on a 6 to 20 Borg scale). Sessional rating of perceived exertion was recorded after each training session ^4^.

**Table S1**: A summary of the exercise intervention

| **Aerobic training** | | | | | **Resistance training** | | | | |
| --- | --- | --- | --- | --- | --- | --- | --- | --- | --- |
| **Microcycle** | **Sessions p/wk.** | **Session type** | **HR range** | **Duration, min** | **Sessions p/wk.** | **Sets** | **Repetitions** | **Rest** | **% RM** |
| **Phase one** | 2 | MCT | 1-20 bpm below VT | 30 | 2 | 2-3 | 10-12 | 45 secs - 1 min | ~60-75% |
| **Phase two** | 2 | MCT  VT or HIIT | 1-20 bpm below VT  VT, or >95% HRpeak | 30  20-25 | 2 | 3-4 | 8-10 | 1-2 min | ~60-85% |
| **Phase three** | 2 | HIIT | >95% HRpeak | 20-25 | 2 | 3 | 10-12 | 45 secs - 1 min | ~60-75% |

Abbreviations: MCT (moderate continuous training), VT (ventilatory threshold), HIIT (high-intensity interval training), HR peak (peak heart rate), RM (maximum repetition).

**Assessment of attendance and compliance with the exercise oncology intervention**

Study investigators recorded exercise session attendance, and the total exercise sessions attended was divided by the total exercise session prescribed. Given the geographical diversity among participants, some participants completed their exercise intervention in a hybrid format. In this case, participants were provided with an online exercise program and the opportunity to complete their supervised exercise intervention via telehealth video conferencing software (health direct Australia, Pty Ltd, Haymarket, Australia).

**Nutritional prehabilitation intervention:**

To meet measured energy and predicted protein requirements via nutrition counselling encompassing high energy high protein diet, food fortification strategies and utilization of oral nutrition support in patients unable to meet their requirements through diet alone. Consideration of enteral nutrition support if oral intake is less than 60% of requirements despite education & supplementation of diet. Immune-Modulating supplementation (e.g., IMPACT ®) shall be prescribed as 3 x 200ml tetra packs daily for five days pre & post-operatively, in accordance with evidence-based practice for the nutritional management of patients undergoing upper GI cancer surgery.^5^

**Psychology support:**

The prehabilitation intervention addressed the barriers and enablers to complying with the three-month exercise intervention in participants who have been either identified by the HADS cut-off or by supervising exercise physiologists monitoring the participants.

**High-Risk Anesthetic Assessment**

A small proportion of high-risk patients account for most of the postoperative morbidity.^6^ The high-risk clinic aims to identify these patients preoperatively and institute strategies to reduce perioperative morbidity and mortality. Specific aims include assessment of perioperative risk; optimization of medical comorbidities, analgesia and functional status; and facilitation of informed decision making and perioperative planning.^7^ This involved identifying goals and values unique to the patient, particularly in the event of planned or unplanned high dependency unit/intensive care admission.

- Feedback to the upper gastrointestinal cancer multidisciplinary team regarding patient safety to proceed through neoadjuvant treatment and surgery.
- Medical optimization of comorbidities: Referral and coordination with relevant specialist physicians at Austin Health
- Smoking cessation
- Optimization of functional status: Coordination with exercise physiologist, nutritionist, psychologist
- Optimization of preoperative analgesia: If chronic pain is present, referral to POPPMED pain clinic at Austin Health. Discussion and planning of perioperative analgesia, including regional/neuraxial blocks.
- Facilitating shared decision making as well as advanced care planning with patients and upper gastrointestinal cancer multidisciplinary team
- Successful smoking cessation

**Outcomes**

Following informed consent, participants were provided with a series of instructions prior to attending each visit throughout the enhanced prehabilitation program. For the physical function assessments, participants were asked to refrain from alcohol, caffeine and moderate to vigorous exercise for 24 hours before all visits.

**Primary outcome**:

- Rate of overall post-operative respiratory complication was defined by the Esophagectomy Complication Consensus Group (ECCG) ^8,9^ (*primary endpoint for power calculation*)

**Secondary outcomes**:

*Surgical and anesthetic outcomes*

- Individual surgical complication rate defined by the ECCG guidelines ^8,9^
- Overall surgical complication rate
- Rate of the highest complication grade as defined by Clavien Dindo classification^10^
- Postoperative length-of-stay (days)
- 30-day hospital readmission rate
- 30-day intensive care unit readmission rate
- 30-day mortality rate
- Rate of achieving textbook outcomes as defined by the Dutch Upper Gastrointestinal Cancer Audit and international consensus guidelines ^11,12^
- Days Alive and at Home within 30 days of surgery (DAH_30_): Total number of days spent at own residence or home within 30 days post-surgery^13^

*Oncological outcomes*

- Rate of starting postoperative chemotherapy
- Rate of completing postoperative chemotherapy

**Physical function assessments**

*Cardiopulmonary exercise test*

A cardiopulmonary exercise test (CPET) was performed to determine peak oxygen uptake (VO_2_peak, ml/kg/min). A progressive ramp protocol was performed on an upright cycle ergometer (Corival V2, Lode BV, Netherlands) with continuous cardiac monitoring 12-lead electrocardiography (Ergocard, Medisoft, Sorrines, Belgium). Expired breath by breath analysis was analyzed using a metabolic measurement system (Ergocard, Medisoft, Sorrines, Belgium). Individual workloads increments (5-25 Watts/minute) were determined by prior pulmonary function test^14^. Tests were initiated with 2 minutes rest followed by 2 minutes unloaded pedaling before initiating stepped workload increases until symptom limitation. Blood pressure was measured every two minutes throughout the CPET ^15^. A plateau in oxygen uptake was used to quantify the achievement of maximal oxygen consumption but in the absence of this, the secondary criteria was used to determine peak effort ^16^. Participants were required to meet at least two of the following criteria: peak effort was considered as follows: >85% of age-predicted maximum heart rate, respiratory exchange ratio >1.10, or volitional fatigue ^16-18^. All CPET’s were supervised by medical staff and testing was ceased in accordance with published criteria^19^. CPET-derived variables included VO_2_peak (L/min), ventilatory threshold (VT), slope in minute ventilation (V_E_) in proportion to the expired volume of carbon dioxide (VCO_2_) (V_E_/VCO_2_), resting systolic and diastolic blood pressure, peak systolic and diastolic blood pressure, resting and peak heart rate, peak power output, respiratory exchange ratio. VT, and VT and V_E_/VCO_2_ slope were calculated using standard guidelines^20^. VO_2_peak was defined as the 30-second moving average of the six highest oxygen consumption values measured over five seconds.

*Functional measure of muscle strength*

Handgrip strength (HGS) was measured by the dietitian via a portable handgrip dynamometer (Saehan Medical, South Korea) and is a validated tool in hospitalized patients to measure muscle strength.^21^ Patients were be asked to perform a maximal contraction with the non-dominant hand on three occasions, with 10 seconds rest between contractions. The maximal score recorded (in kg) and compared to normative grip strength values and established cut-offs for sarcopenia. Handgrip strength (HGS) was measured by a portable handgrip dynamometer (Saehan Medical, South Korea) and is a validated tool in hospitalized patients to measure muscle strength.^21^ Patients were asked to perform a maximal contraction with the non-dominant hand on three occasions, with 10 seconds rest between contractions. The maximal score was recorded (in kg) and compared to normative grip strength values and established cut-offs for sarcopenia.^22^

*30-second sit-to-stand:*

Functional lower limb strength was assessed by the 30-second sit-to-stand test ^23^. The 30-second sit-to-stand test is performed in an armless chair with an appropriate height of 43-46 cm. Participants were instructed to fold their arms across their chest and perform the stand-up and sit-down movements quickly and safely within 30 seconds ^23^. The number of repetitions was recorded after each attempt. Tests were performed in duplicate with adequate rest in between trials.

*Six-minute walk test*

Cardiorespiratory fitness was assessed by the six-minute walk test. Participants were instructed to walk back and forth around a series of cones in an indoor, 30-metre corridor for six minutes ^24^. Before initiating the assessment, resting blood pressure, resting heart rate, oxygen saturation and a rating of perceived exertion were taken. Standardized criteria set by the American Thoracic Society ^24^ were used for this assessment. A stopwatch was used to record the test duration. After the test ceased, the total distance, resting heart rate, blood pressure, rating of perceived exertion and recovery time were monitored.

**Nutritional assessments**

*Diet History*

The dietitian assessed the patient’s food intake via a diet history interview to determine usual food consumption patterns of the individual and detailed information on certain foods. The information collected from the interview was analyzed by the dietitian to calculate the patient’s average daily energy (kJ) and protein (g) intake and compared to the individual measured energy requirement via indirect calorimetry. This information forms the basis of the nutrition intervention with counselling aimed at meeting the individual nutrition requirements. Diet history was administered and performed by a member of the dietetics team.

*Patient-generated Subjective Global Assessment*

Nutritional assessment was by the scored Patient-Generated Subjective Global Assessment (PG-SGA), a validated nutrition assessment tool that allows rapid identification and prioritization of malnutrition in hospitalized patients with cancer.^25^ The PG-SGA is determined from the patient’s nutritional history (weight change, dietary intake and gastrointestinal symptoms), clinical examination (physical signs of malnutrition including depletion of subcutaneous fat stores and muscle mass) and degree of functional impairment. Patients are then classified as well as nourished (SGA A), mild-moderately malnourished (SGA B) or severely malnourished (SGA C), with a numerical score range from 0 (no problems) to 36 (worst problems).

*Anthropometry*

Weight (kg), height (m) and waist circumference (cm) were measured in all patients, and BMI (kg/m^2^) calculated from measured values. Triceps skinfold (TSF) was measured (in mm) using skinfold calipers to measure the fat pad over the left triceps muscle. This is a validated method to assess malnutrition.^26^ Mid-upper arm circumference (MUAC) was measured (in cm) on the left arm at the mid-point between the tip of the shoulder and tip of the elbow (olecranon process and the acromion). The mid-arm muscle area (MAMA) can then be calculated (MAMC = MUAC – π x TSF). The MAMA is a sensitive index of body protein reserve.^26,27^ Calf circumference was measured (in cm) with the participant in the seated position, at the calf’s maximal circumference point on the perpendicular plane to the long axis. Calf circumference is used as a marker for diagnosing low skeletal muscle and sarcopenia.^28^ All anthropometry measures are quick, reproducible bedside measurements were collected by the study dietitian.

**Supplementary Appendix 2**

Baseline comorbidities were quantified using the Charlson Comorbidity Index, which also estimates the probability of survival at 10 years.^29^ Clinicians’ assessment of performance status was classified using the Eastern Cooperative Oncology Group (ECOG) scale.^30^ Clinicians’ assessment of overall surgical fitness were classified using the American Society of Anesthesiologist (ASA) score.^31^ Postoperative medical complications were defined according to the European Perioperative Clinical Outcome definitions.^32^ Postoperative surgical complications were defined by the Esophagectomy Complication Consensus Guidelines.^9^ The severity of each adverse event was classified using the Clavien-Dindo system.^33^ All tumors were staged using the 8^th^ Edition AJCC Cancer Staging Manual.^34^

**Table S2.** Patient satisfaction with multi-modal prehabilitation

| **Patient satisfaction with:** | **Post prehabilitation program** | **Reference** |
| --- | --- | --- |
| Overall care, median (range) | 5 (4-5) | 1: very dissatisfied, 5: very satisfied |
| Exercise therapy, median (range) | 5 (3-5) | 1: very dissatisfied, 5: very satisfied |
| Dietetic service, median (range) | 4 (3-5) | 1: very dissatisfied, 5: very satisfied |
| Psychology service, median (range) | 3 (3-5) | 1: very dissatisfied, 5: very satisfied |
| Medical care, median (range) | 5 (4-5) | 1: very dissatisfied, 5: very satisfied |
| Surgical care, median (range) | 5 (4-5) | 1: very dissatisfied, 5: very satisfied |

Patient satisfaction measured using a 5-point Likert scale: 1: very dissatisfied, 2: below satisfied, 3:

satisfied, 4: above satisfied, 5: very satisfied with their overall care.

**Table S3.** Multivariate predictors of pulmonary complications

| **Characteristics** | **No Pulmonary**  **complications**  **(n=102)** | **Yes Pulmonary**  **complications**  **(n=62)** | **Univariate**  **p-value** | **Multivariate** | |
| --- | --- | --- | --- | --- | --- |
|  |  |  |  | **OR (95% CI)** | **p-value** |
| **Demographics** |  |  |  |  |  |
| Age (years), mean (SD) | 68.7 (9.7) | 66.0 (10.5) | 0.101 | - | - |
| Sex (male), n (%) | 71 (69.2) | 52 (83.9) | 0.043 | 0.65 (0.24-1.71) | 0.858 |
| BMI (kg/m^2^), mean (SD) | 27.6 (5.9) | 26.8 (4.8) | 0.980 | - | - |
| Charlson Co-morbidity Index, score, median (IQR) | 4 (3-6) | 4 (2-6) | 0.201 | - | - |
| Active smoking status at time of surgery, n (%) | 8 (7.8) | 8 (12.9) | 0.294 | - | - |
| ECOG performance status, median (IQR) | 0 (0-1) | 0 (0-0) | 0.224 | - | - |
| **Clinical tumor features** |  |  |  |  |  |
| Histological diagnosis, n (%) |  |  | 0.575 | - | - |
| Adenocarcinoma | 94 (92.2) | 56 (90.3) |  |  |  |
| Adenocarcinoma with squamous differentiation | 1 (1.0) | 0 (0.0) |  |  |  |
| Squamous cell carcinoma | 6 (5.9) | 6 (9.7) |  |  |  |
| Neuroendocrine tumor | 1 (1.0) | 0 (0.0) |  |  |  |
| Anatomical location of tumor, n (%) |  |  | 0.003 | 1.37 (0.33-5.82) | 0.662 |
| Oesophagus/GOJ (Siewert 1 and 2) | 48 (47.1) | 44 (71.0) |  |  |  |
| Stomach/GOJ (Siewert 3) | 54 (52.9) | 18 (29.0) |  |  |  |
| Clinical stage, n (%) |  |  | 0.821 | - | - |
| cT1 N0 M0 | 24 (23.5) | 14 (22.6) |  |  |  |
| cT2-3 N0 M0 | 41 (40.2) | 25 (40.3) |  |  |  |
| cT4a N0 M0 | 2 (2.0) | 2 (3.2) |  |  |  |
| cT4b N0 M0 | 0 (0.0) | 1 (1.6) |  |  |  |
| cT1 N1 M0 | 1 (1.0) | 1 (1.6) |  |  |  |
| cT2-3 N1 M0 | 25 (24.5) | 16 (25.8) |  |  |  |
| cT4a N1 M0 | 9 (8.8) | 3 (4.8) |  |  |  |
| **Pre-operative treatment** |  |  | 0.639 | - | - |
| None, n (%) | 36 (35.3) | 24 (38.7) |  |  |  |
| Chemotherapy (FLOT protocol), n (%) | 59 (57.8) | 32 (51.6) |  |  |  |
| Chemoradiotherapy (CROSS protocol), n (%) | 7 (6.9) | 6 (9.7) |  |  |  |
| **Surgery details** |  |  |  |  |  |
| Type, n (%) |  |  | <0.001 | 0.14 (0.037-0.50) | **0.003** |
| Subtotal gastrectomy | 45 (44.1) | 8 (12.9) |  |  |  |
| Total/Extended total gastrectomy | 17 (16.7) | 17 (27.4) |  |  |  |
| Oesophagectomy | 40 (39.2) | 37 (59.7) |  |  |  |
| Approach, n (%) |  |  | 0.873 | - | - |
| Open | 51 (50.0) | 30 (48.4) |  |  |  |
| Minimally invasive | 51 (50.0) | 32 (51.6) |  |  |  |
| Lymphadenectomy, n (%) |  |  | 0.008 | 4.68 (1.98-12.08) | **0.001** |
| 2-field mediastinal and abdominal | 40 (39.2) | 36 (58.1) |  |  |  |
| D1 abdominal | 10 (9.8) | 0 (0.0) |  |  |  |
| D1+ abdominal | 52 (51.0) | 26 (41.9) |  |  |  |
| Lymph node yield, median (IQR) | 33 (23-47) | 32 (23-45) | 0.992 | - | - |
| Intraoperative complications, n (%) | 3 (2.9) | 3 (4.8) | 0.674 | - | - |
| Surgical duration, minutes, mean (SD) | 352.0 (116.5) | 435.6 (129.3) | <0.001 | 1.01 (1.002-1.01) | **0.009** |
| Epidural anesthesia use, n (%) | 36 (35.3) | 34 (54.8) | 0.016 | 1.82 (0.88-3.81) | 0.110 |
| **Prehabilitation program, n (%)** | 34 (33.3) | 9 (14.5) | 0.010 | 0.22 (0.08-0.55) | **0.002** |

ASA: American Society of Anesthesiologists, BMI: Body mass index, CI: confidence interval, ECOG: Eastern Cooperative Oncology Group performance status grading, GOJ: Gastroesophageal junction, IQR: Interquartile range, SD: Standard deviation.

**Table S4**. Outcomes of patients who underwent minimally invasive surgery.

| **Outcomes** | **Control**  **(n=47)** | **Prehabilitation**  **(n=36)** | **OR**  **(95% CI)** | **p-value** |
| --- | --- | --- | --- | --- |
| **Perioperative outcomes** |  |  |  |  |
| Duration of ICU stay, days, median (IQR) | 1 (0-7) | 1 (1-3) | - | **0.042** |
| Duration of hospital stay, days, median (IQR) | 13 (8-26) | 11 (8-16) | - | **0.009** |
| 30-day reintervention, n (%) | 12 (25.5) | 10 (27.8) | 1.12 (0.45-3.09) | 1.000 |
| 30-day ICU readmission, n (%) | 10 (21.3) | 6 (16.7) | 0.74 (0.23-2.22) | 0.780 |
| 30-day hospital readmission, n (%) | 3 (6.4) | 3 (8.3) | 1.33 (0.30-5.98) | 1.000 |
| 30-day mortality, n (%) | 2 (4.3) | 0 (0.0) | 0.00 (0.00-2.81) | 0.503 |
| Achieved textbook outcomes, n (%) | 19 (40.4) | 17 (47.2) | 1.32 (0.53-1.87) | 0.656 |
| DAH30, days, median (IQR) | 17 (3-22) | 18 (13-22) | - | 0.064 |
| Received adjuvant treatment, n (%) * | 15 (45.5) | 22 (81.5) | 5.28 (1.70-15.53) | **0.007** |
| **Clavien-Dindo complication grade, n (%)** |  |  |  | **0.020** |
| No complications | 13 (12.7) | 20 (32.3) |  |  |
| Grade 1 | 6 (5.9) | 2 (3.2) |  |  |
| Grade 2 | 14 (13.7) | 2 (3.2) |  |  |
| Grade 3 a/b | 7 (6.9) | 8 (12.9) |  |  |
| Grade 4 a/b | 5 (4.9) | 4 (6.5) |  |  |
| Grade 5 | 2 (2.0) | 0 (0.0) |  |  |
| **Pulmonary, n (%)** |  |  |  |  |
| All pulmonary complications | 25 (53.2) | 7 (19.4) | 0.21 (0.09-0.61) | **0.003** |
| Pneumonia | 17 (36.2) | 3 (8.3) | 0.16 (0.05-0.54) | **0.004** |
| Atelectasis requiring intervention | 11 (23.4) | 0 (0.0) | 0.00 (0.00-0.34) | **0.002** |
| Pleural effusion requiring drainage | 6 (12.8) | 2 (5.6) | 0.40 (0.08-1.77) | 0.456 |
| Respiratory failure requiring intubation | 4 (8.5) | 0 (0.0) | 0.00 (0.00-1.32) | 0.129 |
| Pneumothorax requiring intervention | 3 (6.4) | 0 (0.0) | 0.00 (0.00-1.48) | 0.254 |
| Acute respiratory distress syndrome | 2 (4.3) | 0 (0.0) | 0.00 (0.00-2.81) | 0.503 |
| Acute aspiration | 5 (10.6) | 2 (5.6) | 0.49 (0.09-2.59) | 0.693 |
| Tracheobronchial injury | 2 (4.3) | 0 (0.0) | 0.00 (0.00-2.81) | 0.503 |
| **Cardiac, n (%)** |  |  |  |  |
| All cardiac complications | 14 (29.8) | 2 (5.6) | 0.14 (0.03-0.62) | **0.006** |
| Arrhythmias requiring intervention | 11 (23.4) | 2 (5.6) | 0.19 (0.04-0.94) | **0.034** |
| Congestive heart failure requiring intervention | 5 (10.6) | 0 (0.0) | 0.00 (0.00-0.86) | **0.044** |
| Myocardial infarction | 1 (2.1) | 0 (0.0) | 0.00 (0.00-11.75) | 1.000 |
| **Gastrointestinal, n (%)** |  |  |  |  |
| Anastomotic leak | 6 (12.8) | 2 (5.6) | 0.40 (0.08-1.77) | 0.270 |
| Conduit necrosis | 1 (2.1) | 0 (0.0) | 0.00 (0.00-11.75) | 1.000 |
| Ileus delaying enteral feeding | 4 (8.5) | 7 (19.4) | 2.56 (0.70-8.40) | 0.196 |
| Small bowel obstruction | 2 (4.3) | 2 (5.6) | 1.32 (0.20--8.75) | 1.000 |
| Chyle leak |  |  |  |  |
| Pancreatitis | 1 (2.1) | 1 (2.8) | 1.31 (0.07-25.45) | 1.000 |
| Liver dysfunction | 5 (10.6) | 0 (0.0) | 0.00 (0.00-0.86) | **0.044** |
| Acute diaphragmatic hernia | 0 (0.0) | 1 (2.8) | 0.00 (0.00-6.89) | 0.434 |
| **Infection, n (%)** |  |  |  |  |
| General sepsis | 2 (4.3) | 1 (2.8) | 0.64 (0.04-5.73) | 1.000 |
| Surgical site infection requiring intervention | 3 (6.4) | 1 (2.8) | 0.42 (0.03-2.94) | 0.629 |
| Line infection requiring intervention | 1 (2.1) | 0 (0.0) | 0.00 (0.00-11.75) | 1.000 |
| Intrathoracic abscess | 2 (4.3) | 3 (8.3) | 2.05 (0.40-11.92) | 0.648 |
| Intraabdominal abscess | 3 (6.4) | 1 (2.8) | 0.42 (0.03-2.94) | 0.629 |
| **Neurological, n (%)** |  |  |  |  |
| Delirium | 4 (8.5) | 1 (2.8) | 0.31 (0.02-2.03) | 0.382 |
| **Hematological, n (%)** |  |  |  |  |
| Bleeding requiring intervention | 2 (4.3) | 0 (0.0) | 0.00 (0.00-2.81) | 0.503 |
| Venous thromboembolism | 3 (6.4) | 2 (5.6) | 0.86 (0.14-4.42) | 1.000 |
| **Urological, n (%)** |  |  |  |  |
| Acute kidney injury | 5 (10.6) | 0 (0.0) | 0.00 (0.00-0.86) | **0.044** |
| Urinary tract infection | 1 (2.1) | 0 (0.0) | 0.00 (0.00-11.75) | 1.000 |
| **Skin, n (%)** |  |  |  |  |
| Wound dehiscence | 1 (2.1) | 0 (0.0) | 0.00 (0.00-11.75) | 1.000 |
| Acute abdominal wall hernia | 0 (0.0) | 1 (2.8) | 0.00 (0.00-6.89) | 0.434 |

CI: confidence interval, IQR: Interquartile range, OR: Odds ratio. *Clinically indicated to receive adjuvant treatment (Control n=33, SOCS Prehab n=27)

**Figure S1.** Complication profile of patients with Grade ≥3 Clavien-Dindo complication grade

**References**

1.	Campbell KL, Winters-Stone KM, Wiskemann J, et al. Exercise guidelines for cancer survivors: consensus statement from international multidisciplinary roundtable. *Med Sci Sports Exerc.* 2019;51(11):2375-2390.

2.	Howden EJ, Sarma S, Lawley JS, et al. Reversing the cardiac effects of sedentary aging in middle age-a randomized controlled trial: implications for heart failure prevention. *Circulation.* 2018;137(15):1549-1560.

3.	Iwasaki K, Zhang R, Zuckerman JH, Levine BD. Dose-response relationship of the cardiovascular adaptation to endurance training in healthy adults: how much training for what benefit? *J Appl Physiol (1985).* 2003;95(4):1575-1583.

4.	Bigaran A, Howden EJ, Foulkes S, et al. Prescribing Exercise in Early-Stage Breast Cancer During Chemotherapy: A Simple Periodized Approach to Align With the Cyclic Phases of Chemotherapy. *J Strength Cond Res.* 2022;36(10):2934-2941.

5.	Weimann A, Braga M, Carli F, et al. ESPEN practical guideline: Clinical nutrition in surgery. *Clin Nutr.* 2021;40(7):4745-4761.

6.	Pearse RM, Harrison DA, James P, et al. Identification and characterisation of the high-risk surgical population in the United Kingdom. *Crit Care.* 2006;10(3):R81.

7.	Whiteman AR, Dhesi JK, Walker D. The high-risk surgical patient: a role for a multi-disciplinary team approach? *Br J Anaesth.* 2016;116(3):311-314.

8.	van der Werf LR, Busweiler LAD, van Sandick JW, van Berge Henegouwen MI, Wijnhoven BPL, Dutch Upper GICAg. Reporting National Outcomes After Esophagectomy and Gastrectomy According to the Esophageal Complications Consensus Group (ECCG). *Ann Surg.* 2020;271(6):1095-1101.

9.	Low DE, Alderson D, Cecconello I, et al. International Consensus on Standardization of Data Collection for Complications Associated With Esophagectomy: Esophagectomy Complications Consensus Group (ECCG). *Ann Surg.* 2015;262(2):286-294.

10.	Clavien PA, Barkun J, de Oliveira ML, et al. The Clavien-Dindo classification of surgical complications: five-year experience. *Ann Surg.* 2009;250(2):187-196.

11.	Busweiler LA, Schouwenburg MG, van Berge Henegouwen MI, et al. Textbook outcome as a composite measure in oesophagogastric cancer surgery. *Br J Surg.* 2017;104(6):742-750.

12.	Kalff MC, van Berge Henegouwen MI, Gisbertz SS. Textbook outcome for esophageal cancer surgery: an international consensus-based update of a quality measure. *Dis Esophagus.* 2021;34(7).

13.	Myles PS, Shulman MA, Heritier S, et al. Validation of days at home as an outcome measure after surgery: a prospective cohort study in Australia. *BMJ Open.* 2017;7(8):e015828.

14.	Pretto JJ, Braun GW, Guy PA. Using baseline respiratory function data to optimize cycle exercise test duration. *Respirology (Carlton, Vic).* 2001;6(4):287-291.

15.	Balady GJ, Arena R, Sietsema K, et al. Clinician's Guide to cardiopulmonary exercise testing in adults: a scientific statement from the American Heart Association. *Circulation.* 2010;122(2):191-225.

16.	American Thoracic S, American College of Chest P. ATS/ACCP Statement on cardiopulmonary exercise testing. *Am J Respir Crit Care Med.* 2003;167(2):211-277.

17.	Jones LW, Eves ND, Haykowsky M, Joy AA, Douglas PS. Cardiorespiratory exercise testing in clinical oncology research: systematic review and practice recommendations. *The Lancet Oncology.* 2008;9(8):757-765.

18.	Balady GJ, Arena R, Sietsema K, et al. Clinician's Guide to cardiopulmonary exercise testing in adults: a scientific statement from the American Heart Association. *Circulation.* 2010;122(2):191-225.

19.	American College of Sports Medicine. *ACSM's Guidelines for Exercise Testing and Prescription.* 8th ed. Baltimore, Maryland, USA: Lippincott Williams & Wilkins; 2014.

20.	Bard RL, Gillespie BW, Clarke NS, Egan TG, Nicklas JM. Determining the best ventilatory efficiency measure to predict mortality in patients with heart failure. *J Heart Lung Transplant.* 2006;25(5):589-595.

21.	Cruz-Jentoft AJ, Bahat G, Bauer J, et al. Sarcopenia: revised European consensus on definition and diagnosis. *Age Ageing.* 2019;48(1):16-31.

22.	Muscular weakness assessment: use of normal isometric strength data. The National Isometric Muscle Strength (NIMS) Database Consortium. *Arch Phys Med Rehabil.* 1996;77(12):1251-1255.

23.	Pavasini R, Guralnik J, Brown JC, et al. Short Physical Performance Battery and all-cause mortality: systematic review and meta-analysis. *BMC medicine.* 2016;14(1):215.

24.	Laboratories ATSCoPSfCPF. ATS statement: guidelines for the six-minute walk test. *American journal of respiratory and critical care medicine.* 2002;166(1):111-117.

25.	Bauer J, Capra S, Ferguson M. Use of the scored Patient-Generated Subjective Global Assessment (PG-SGA) as a nutrition assessment tool in patients with cancer. *Eur J Clin Nutr.* 2002;56(8):779-785.

26.	Yin L, Fan Y, Lin X, et al. Fat mass assessment using the triceps skinfold thickness enhances the prognostic value of the Global Leadership Initiative on Malnutrition criteria in patients with lung cancer. *Br J Nutr.* 2022;127(10):1506-1516.

27.	Burden ST, Stoppard E, Shaffer J, Makin A, Todd C. Can we use mid upper arm anthropometry to detect malnutrition in medical inpatients? A validation study. *J Hum Nutr Diet.* 2005;18(4):287-294.

28.	Gonzalez MC, Mehrnezhad A, Razaviarab N, Barbosa-Silva TG, Heymsfield SB. Calf circumference: cutoff values from the NHANES 1999-2006. *Am J Clin Nutr.* 2021;113(6):1679-1687.

29.	Charlson ME, Pompei P, Ales KL, MacKenzie CR. A new method of classifying prognostic comorbidity in longitudinal studies: development and validation. *J Chronic Dis.* 1987;40(5):373-383.

30.	Oken MM, Creech RH, Tormey DC, et al. Toxicity and response criteria of the Eastern Cooperative Oncology Group. *Am J Clin Oncol.* 1982;5(6):649-655.

31.	Doyle DJH, J.M.; Garmon, E.H. *Society of Anesthesiologists Classification.* StatPearls Publishing LLC; 2023.

32.	Jammer I, Wickboldt N, Sander M, et al. Standards for definitions and use of outcome measures for clinical effectiveness research in perioperative medicine: European Perioperative Clinical Outcome (EPCO) definitions: a statement from the ESA-ESICM joint taskforce on perioperative outcome measures. *Eur J Anaesthesiol.* 2015;32(2):88-105.

33.	Dindo D, Demartines N, Clavien PA. Classification of surgical complications: a new proposal with evaluation in a cohort of 6336 patients and results of a survey. *Ann Surg.* 2004;240(2):205-213.

34.	Amin MB, Greene FL, Edge SB, et al. The Eighth Edition AJCC Cancer Staging Manual: Continuing to build a bridge from a population-based to a more "personalized" approach to cancer staging. *CA Cancer J Clin.* 2017;67(2):93-99.
